# Manipulating of P2/O3 Composite Sodium Layered Oxide Cathode through Ti Substitution and Synthesis Temperature

**DOI:** 10.3390/nano13081349

**Published:** 2023-04-12

**Authors:** Xiaobai Ma, Hao Guo, Jianxiang Gao, Xufeng Hu, Zhengyao Li, Kai Sun, Dongfeng Chen

**Affiliations:** China Institute of Atomic Energy, Beijing 102413, China

**Keywords:** P2/O3 composite, layered oxide cathode, synergistic effect, Na_0.8_Ni_0.4_Mn_0.4_Ti_0.2_O_2_

## Abstract

P2/O3 composite sodium layered oxide has emerged as a promising cathode for high-performance Na-ion batteries. However, it has been challenging to regulate accurately the phase ratio of P2/O3 composite due to their high compositional diversity, which brings about some difficulty in manipulating the electrochemical performance of P2/O3 composite. Here, we explore the effect of Ti substitution and the synthesis temperature on the crystal structure and Na storage performance of Na_0.8_Ni_0.4_Mn_0.6_O_2_. The investigation indicates Ti-substitution and altering synthesis temperature can rationally manipulate the phase ratio of P2/O3 composite, thereby purposefully regulating the cycling and rate performance of P2/O3 composite. Typically, O3-rich Na_0.8_Ni_0.4_Mn_0.4_Ti_0.2_O_2_-950 shows excellent cycling stability with a capacity retention of 84% (3C, 700 cycles). By elevating the proportion of P2 phase, Na_0.8_Ni_0.4_Mn_0.4_Ti_0.2_O_2_-850 displays concurrently improved rate capability (65% capacity retention at 5 C) and comparable cycling stability. These findings will help guide the rational design of high-performance P2/O3 composite cathodes for sodium-ion batteries.

## 1. Introduction

Na-ion batteries (SIBs) have received wide attention and are considered a promising alternative to Li-ion batteries (LIBs) in the large-scale energy storage field due to the abundance and even distribution of sodium resources [1,2,3,4,5]. Although the knowledge and experience obtained from current LIBs can be applied to the development of SIB, the larger size of Na^+^ compared to Li^+^ results in cathode materials that are prone to suffer from detrimental phase transitions and structural degradation [6,7,8]. Therefore, it is still a great challenge to develop cathodes with high reversible capacity, excellent cycling stability, and rate capability. Up to now, a variety of cathodes, including layered transition metal oxides [9,10], polyanionic compounds [11,12], and Prussian blue analogues [13,14], have been proposed and investigated. Layered transition metal oxides, Na_x_TMO_2_ (TM = transition metals), are promising cathodes owing to their high theoretical capacity and easy fabrication process. According to the positions occupied by Na ions and the number of TMO_2_ layers in the unit cell, Delmas et al. classified these Na_x_TMO_2_ into two main types (O3, P2), where the letter P or O and the number 2 or 3 represent the coordination environment of Na (P: trigonal prismatic and O: octahedral sites) and the number of repeated TM layers in the unit cell, respectively [15]. It is well acknowledged that a low Na content (x < 0.7) enables the formation of a P2-type structure, while the O3-type structure has a high Na content (x > 0.8) [16]. It is worth noting that the diffusion of Na ions in O3-type structures from one octahedral site to an adjacent one needs to pass through the intermediate tetrahedral site that imposes a high energy barrier for Na diffusion, which results in most O3-type cathodes exhibiting low ionic diffusivity and poor rate capability [17,18]. For the P2-type structure, direct Na ion diffusion between neighboring prismatic sites is feasible, supporting the fast migration of Na ions and therefore improved rate performance [19]. Nevertheless, the low Na content in the P2-type structure causes a low initial capacity and irreversible P2-O2 phase transition with a large volume change at high voltage, which limits its application in the full cell. Different strategies, such as doping/substitution [20,21,22,23,24], coating [25], and microstructure design [26] have been adopted to address these issues. Although considerable progress from both engineering and scientific points of view has been obtained in the last decade [27,28,29], it is difficult to completely solve the inherent disadvantages of single-phase structures. Recently, multiphase cathodes combining the advantages of different single-phase structures and overcoming their disadvantages have gained wide attention because of the synergetic effect [30,31]. One feasible strategy for designing P2/O3 composite is to modify the composition of the transition metal (TM) layer. For example, Guo et al. reported that the biphasic Na_0.66_Li_0.18_Mn_0.71_Ni_0.21_Co_0.08_O_2+δ_ can deliver a high discharge specific capacity of 200 mAh/g at a 0.1 C rate with good cycling performance [32]. Qi et al. prepared a series of Na_x_[Ni_0.2_Fe_x-0.4_Mn_1.2-x_]O_2_ samples by adjusting the composition of Na, Mn, and Fe. They found the hybrid structure of Na_0.78_Ni_0.2_Fe_0.38_Mn_0.42_O_2_ deliver a discharge-specific capacity of 86 mAh/g with excellent high-rate performance and cycling life [33]. Another practicable strategy for fabricating the P2/O3 composite is surface modification and morphology design, which integrate the P2 and O3 phases into a hierarchical shell structure. For example, Sun et al. designed a heterostructure consisting of P2-Na_2/3_MnO_2_—coated O3-NaNi_0.5_Mn_0.5_O_2_. They pointed out that enhanced Na storage performance of the heterostructure is attributed to the synergistic effect of the biphasic structure, in which the O3 phase core provides a sufficient Na reservoir while the P2-type structure services as a protective layer [34]. It has been demonstrated that the P2/O3 composite obtained by element substitution and microstructure control shows better comprehensive performance than single-phase structures [35,36,37,38]. Nevertheless, it is still difficult to improve capacity and cycling stability simultaneously, indicating that further component optimization is necessary. In addition, the underlying influence of synthesis temperature on the phase structure and electrochemical performance remains to be unveiled.

It is well known that when sodium stoichiometry is moderate (0.7~0.8), synthesis temperature and element compositions in the TM layer have a significant effect on the phase ratio of the P2/O3 composite, thereby providing tunable electrochemical performance. In this work, we used Na_0.8_Ni_0.4_Mn_0.6_O_2_ as a starting model. Then, we investigated the effect of Ti substitution and synthesis temperature (from 850 to 1000 °C) on the crystal structure and Na storage performance of Na_0.8_Ni_0.4_Mn_0.6_O_2_ and provided a rational guideline for the modulation of phase structure in P2/O3 composite. X-ray diffraction (XRD) and transmission electron microscopy (TEM) data demonstrate that Ti substitution and high synthesis temperature enable the formation of the O3-type structure while suppressing the appearance of the P2-type structure. The biphasic Na_0.8_Ni_0.4_Mn_0.4_Ti_0.2_O_2_-950 with O3-rich characteristics exhibits a specific capacity of 120 mAh/g and outstanding long-cycle stability, with 84% capacity retention at 3 C after 700 cycles. The biphasic Na_0.8_Ni_0.4_Mn_0.4_Ti_0.2_O_2_-850 with elevated P2 characteristics shows similar capacity, comparable cycling stability, and better rate performance (65% capacity retention at 5 C rate) than Na_0.8_Ni_0.4_Mn_0.4_Ti_0.2_O_2_-950. Ex situ X-ray diffraction results indicate that the Na_0.8_Ni_0.4_Mn_0.4_Ti_0.2_O_2_-950 cathode displays a high reversible phase transition of P2/O3-P2/P3. The excellent comprehensive performance of the P2/O3 composite makes it a promising cathode for SIBs.

## 2. Materials and Methods

### 2.1. Material Preparation

Na_0.8_Ni_0.4_Mn_0.6-x_Ti_x_O_2_-950 (x = 0, 0.2, 0.4, and 0.6, denoted as 846-Mn-950, 8442-950, 8424-950, and 846-Ti-950, respectively) and Na_0.8_Ni_0.4_Mn_0.4_Ti_0.2_O_2_-T (T = 850, 900, 950, and 1000 °C, denoted as 8442-T) samples were synthesized by a solid-state method using Na_2_CO_3_ (99%), NiO (99%), MnO_2_ (98%), and TiO_2_ (99%) as starting materials. An excess of 2 mol% of Na_2_CO_3_ was used. The precursors were mixed in an agate mortar for 30 min and further pressed into pellets for calcination. Then Na_0.8_Ni_0.4_Mn_0.6-x_Ti_x_O_2_ pellets were fired at 950 °C for 15 h in a muffle furnace with a 5 °C/min rate under air and cooled to room temperature naturally. Na_0.8_Ni_0.4_Mn_0.4_Ti_0.2_O_2_-T pellets were fired at different temperatures (850, 900, 950, and 1000 °C) for 15 h in a muffle furnace with a 5 °C/min rate under air and cooled to room temperature naturally. All the above chemicals were purchased from Alfa Aesar Company and were used without any further purification. All the as-prepared materials were stored in an Ar-filled glove box.

### 2.2. Structure Characterization

The elemental compositions of Na_0.8_Ni_0.4_Mn_0.4_Ti_0.2_O_2_-T materials were analyzed by inductively coupled plasma-atomic emission spectroscopy (ICP-AES, Agilent, ICPOES730). X-ray diffraction data were collected on an X-ray diffractometer (D8 Bruker) using Cu Kα radiation (λ_1_ = 1.540 Å, λ_2_ = 1.544 Å) in a scanning range (2θ) of 10–70°. XRD data were refined by using FullProf software based on the Rietveld method. The morphology of Na_0.8_Ni_0.4_Mn_0.4_Ti_0.2_O_2_-T samples was characterized by a scanning electron microscope (Hitachi-S4800). Transmission electron microscopy (TEM) images and energy dispersive X-ray spectroscopy (EDS) mappings were obtained by using a JEOL ARM 300F microscope. In the ex-situ XRD investigation, electrodes were charged to different voltages versus Na metal. Then electrodes were disassembled in an Ar-filled glovebox. The obtained cathodes were washed with dimethyl carbonate before XRD measurement.

### 2.3. Electrochemical Measurement

These working electrodes were fabricated by mixing active materials with carbon nanotube and polytetrafluoroethylene at a weight ratio of 80:15:5. Then the components were rolled into thin films with a loading mass of ~4 mg/cm^2^. The coin-type cells (CR2032) were assembled in an argon-filled glove box, using glass fiber as the separator, 1.0 M NaClO_4_/propylene carbonate/ethylene carbonate (PC:EC = 1:1 in volume), 5% fluoroethylene carbonate (FEC) in volume, and Na foil as the electrolyte and counter electrode, respectively. The galvanostatic charge and discharge measurements of cells (1 C = 160 mA/g) were carried out between 2.0 and 4.2 V on a Neware battery cycler (CT-4008T-5V10mA-164, Shenzhen, China) test system. Cyclic voltammetry (CV) was carried out at a scan rate of 0.1 mV/s on a CHI660E Electrochemical Workbench (Shanghai, China).

## 3. Results and Discussion

### 3.1. Structural Analysis

**Figure 1:**
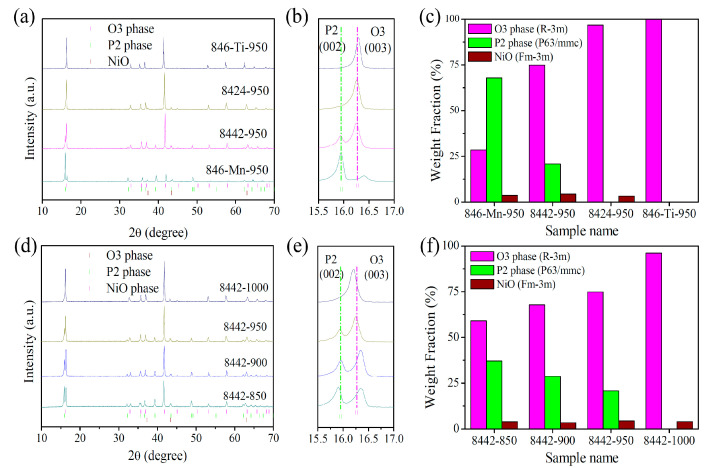
(**a**) XRD patterns of 846-Mn-950, 8442-950, 8424-950, and 846-Ti-950 samples; (**b**) corresponding partial enlargement; (**c**) weight fraction of O3, P2, and NiO phases. (**d**) XRD patterns of 8442-850, 8442-900, 8442-950, and 8442-1000 samples; (**e**) corresponding partial enlargement; (**f**) weight fraction of O3, P2, and NiO phases.

To investigate the effect of Ti-substitution on the crystal structure of Na_0.8_Ni_0.4_Mn_0.6_O_2_, we prepared a series of Na_0.8_Ni_0.4_Mn_0.6-x_Ti_x_O_2_-950 (x = 0, 0.2, 0.4, and 0.6, denoted as 846-Mn-950, 8442-950, 8424-950, and 846-Ti-950, respectively) samples using a solid-state method as described in the Section 2. As shown in Figure 1a,b, Na_0.8_Ni_0.4_Mn_0.6-x_Ti_x_O_2_-950 samples show a phase transition from P2-rich P2/O3 composite structure to single-phase O3-type structure with the increase in Ti content, which demonstrates Ti substitution enables the formation of the O3 phase and suppresses the appearance of the P2 phase. To certify the phase ratio of P2/O3, we refined the XRD data of Na_0.8_Ni_0.4_Mn_0.6-x_Ti_x_O_2_-950 using the Rietveld method, and the corresponding refinement results were displayed in Figure S1 and Tables S1–S5. It can be seen from Figure 1c that the prepared material P2/O3-Na_0.8_Ni_0.4_Mn_0.6_O_2_-950 is composed of 67.8 wt% P2 phase, 28.5 wt% O3 phase, and 3.7 wt% NiO. Ti-substitution results in a Na_0.8_Ni_0.4_Mn_0.4_Ti_0.2_O_2_-950 sample demonstrate an O3-rich characteristic (20.8 wt% P2 phase, 74.9 wt% O3 phase, and 4.3 wt% NiO). When the content of substituted Ti exceeds 0.4, the P2 phase disappears and only the O3 phase is present. Subsequently, we selected P2/O3 composite Na_0.8_Ni_0.4_Mn_0.4_Ti_0.2_O_2_-950 as the initial material to further study the effect of synthesis temperature on the phase structure of Na_0.8_Ni_0.4_Mn_0.4_Ti_0.2_O_2_-T (T = 850, 900, 950, and 1000 °C, denoted as 8442-T) samples. As expected, Na_0.8_Ni_0.4_Mn_0.4_Ti_0.2_O_2_-T samples show a gradual phase change behavior with the increasing synthesis temperature. It can be found from XRD patterns and refinement results that low temperature leads to an increase in the P2 phase proportion, while high temperature facilitates an increase in the O3 phase proportion (Figure 1d–f and Figure S2, and Tables S6–S9). These results indicate the phase ratio of P2/O3 can be rationally manipulated via adjusting Ti content and synthesis temperature, which will make a difference to the cycling life and rate capability of the P2/O3 composite.

**Figure 2:**
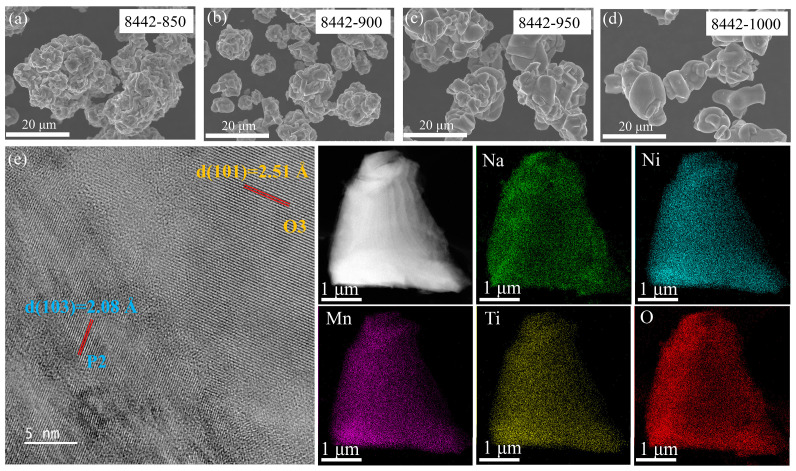
Morphology of (**a**) 8442-850, (**b**) 8442-900, (**c**) 8442-950, and (**d**) 8442-1000, respectively. (**e**) HRTEM image and EDS mapping of 8442-950 sample.

The overall compositions of Na_0.8_Ni_0.4_Mn_0.4_Ti_0.2_O_2_-T materials, which are confirmed by inductively coupled plasma-atomic emission spectroscopy (ICP-AES), are consistent with the designed components (Table S10), demonstrating that high synthesis temperatures result in the decrease in Na content in Na_0.8_Ni_0.4_Mn_0.4_Ti_0.2_O_2_-T materials due to the evaporation of Na_2_CO_3_. The morphology of as-prepared Na_0.8_Ni_0.4_Mn_0.4_Ti_0.2_O_2_-T samples was characterized by scanning electron microscopy (SEM). As depicted in Figure 2a–d, the as-prepared 8442-850 sample is composed of blocky-shaped particles, where the surfaces involve the agglomeration of primary particles. With the increase in temperature, the agglomeration phenomenon of 8442-T samples was relieved, and the surfaces became denser and smoother. The particle size distribution of all as-prepared 8442-T samples is in the range of 5–20 μm. We selected the P2/O3 biphasic 8442-950 sample as an example and further investigated the detailed crystal structure and element distribution by high-resolution transmission electron microscopy (HRTEM). Based on the results of HRTEM (Figure 2e), two kinds of lattice fringes with different interplanar distances (d spacing values) are 2.51 and 2.08 Å, which correspond to the (101) and (103) planes of O3 and P2 structures, respectively, and are compatible with the results of XRD refinement. In addition, energy dispersive X-ray spectroscopy (EDS) elementary mappings (Figure 2e) indicate the Na, Ni, Mn, Ti, and O elements are uniformly distributed in the as-prepared 8442-950 sample.

### 3.2. Electrochemical Performance

**Figure 3:**
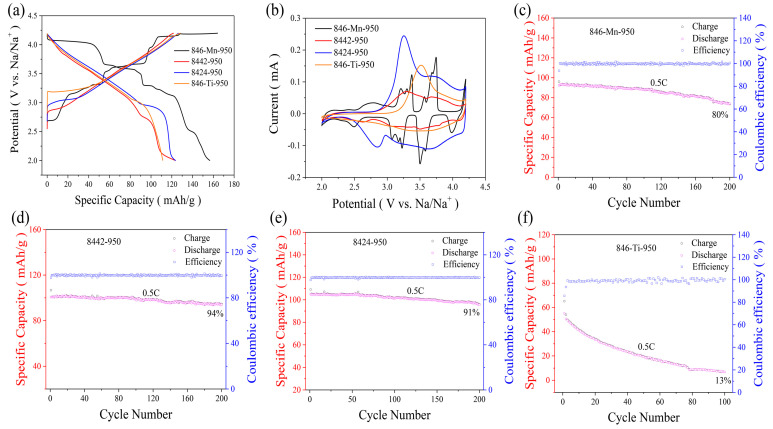
(**a**) charge-discharge curves of Na_0.8_Ni_0.4_Mn_0.6-x_Ti_x_O_2_-950 electrodes at 0.1 C between 2.0 and 4.2 V. (**b**) cyclic voltammetry (CV) profiles of Na_0.8_Ni_0.4_Mn_0.6-x_Ti_x_O_2_-950 electrodes scanned at a rate of 0.1 mV/s. (**c**–**f**) cycling performance of Na_0.8_Ni_0.4_Mn_0.6-x_Ti_x_O_2_-950 electrodes at 0.5 C rate.

In order to understand the effect of Ti-substitution on electrochemical performance, Na_0.8_Ni_0.4_Mn_0.6-x_Ti_x_O_2_-950 electrodes were tested by using coin-type half cells. As shown in Figure 3a, the P2-rich Na_0.8_Ni_0.4_Mn_0.6_O_2_-950 cathode shows a step-like voltage curve associated with Na^+^/vacancy ordering and P2-O2 phase transition, which is consistent with the previously reported P2-type cathodes. This phenomenon is also observed in the cyclic voltammetry (CV) curves of Na_0.8_Ni_0.4_Mn_0.6_O_2_-950 (Figure 3b). Multiple pairs of anodic/cathodic peaks at 2.76/2.49, 3.21/3.07, 3.30/3.17, 3.37/3.22, 3.74/3.49, and 4.20/4.00 V can be observed in the CV curves of the Na_0.8_Ni_0.4_Mn_0.6_O_2_-950 cathode, corresponding to multiple voltage plateaus in charge-discharge curves. Although Na_0.8_Ni_0.4_Mn_0.6_O_2_-950 delivers a reversible specific capacity of 158 mAh/g in the voltage range of 2.0–4.2 V at 0.1 C (16 mA/g), the cycling stability is unsatisfactory. With the increased Ti substitution, the charge-discharge curves become smoother, and the high-voltage plateau disappears, which can also be reflected by the corresponding CV curves. As a result, O3-rich Na_0.8_Ni_0.4_Mn_0.4_Ti_0.2_O_2_-950 shows excellent cycling stability with a capacity retention of 94% (200 cycles, 0.5 C), which is much better than that of P2-rich Na_0.8_Ni_0.4_Mn_0.6_O_2_-950 (80%). It is noteworthy that further Ti-substitution will reduce the cycling stability, indicating moderate Ti-substitution is feasible to improve the electrochemical property.

**Figure 4:**
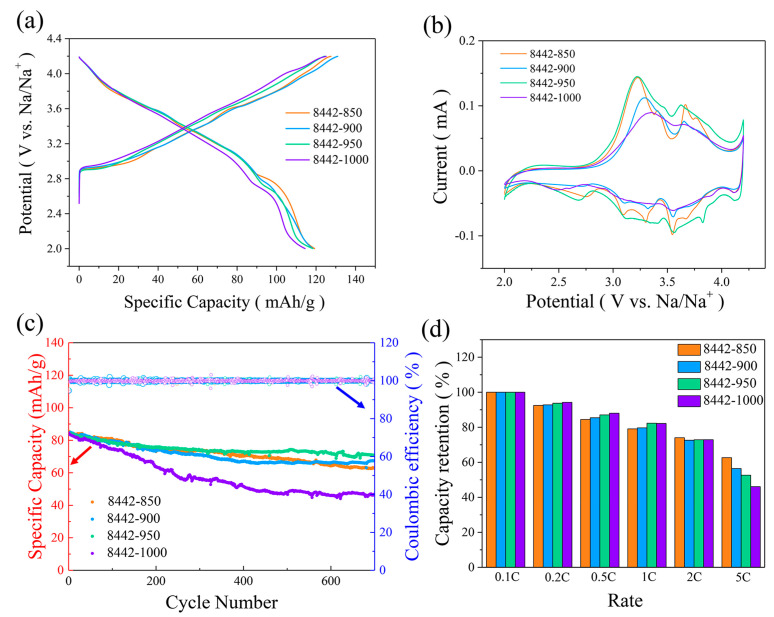
(**a**) charge-discharge curves of Na_0.8_Ni_0.4_Mn_0.4_Ti_0.2_O_2_-T electrodes at 0.1 C between 2.0 and 4.2 V. (**b**) cyclic voltammetry (CV) profiles of Na_0.8_Ni_0.4_Mn_0.4_Ti_0.2_O_2_-T electrodes scanned at a rate of 0.1 mV/s. (**c**) cycling performance and (**d**) rate capability of Na_0.8_Ni_0.4_Mn_0.4_Ti_0.2_O_2_-T electrodes.

The above-mentioned XRD results indicate the phase ratio of the P2/O3 composite can be adjusted by altering the synthesis temperature. To study the influence of synthesis temperature on cycling stability and rate performance of Na_0.8_Ni_0.4_Mn_0.4_Ti_0.2_O_2_, we performed galvanostatic charge/discharge and cyclic voltammetry measurements using half-cells. It can be found from Figure 4a,b that O3-rich 8442-850, 8442-900, and 8442-950 cathodes show similar charge-discharge profiles and cyclic voltammetry curves, which are different compared with those of the single-phase O3-type 8442-1000 cathode. Due to the synergistic effect of P2/O3 phases, O3-rich 8442-850, 8442-900, and 8442-950 cathodes exhibit much better cycling stability and higher specific capacities than O3-type 8442-1000 (Figure 4c). It should be pointed out that 8442-950, with the highest O3-phase ratio (20.8 wt% P2 phase, 74.9 wt% O3 phase), has the best cycling performance with a capacity retention of 84% (700 cycles, 3 C), which is also better than those of previously reported P2/O3 composite materials (Table S11). The rate capabilities of Na_0.8_Ni_0.4_Mn_0.4_Ti_0.2_O_2_-950 cathodes at rates of 0.1 C, 0.2 C, 0.5 C, 1 C, 2 C, and 5 C were also examined and displayed in Figure S3a–d. To conveniently compare the rate retention, the capacities at different rates were normalized with reference to the values obtained at 0.1 C (Figure 4d). It can be found that Na_0.8_Ni_0.4_Mn_0.4_Ti_0.2_O_2_-850 cathodes show better rate performance than other cathodes at high rates. These results demonstrate O3-rich Na_0.8_Ni_0.4_Mn_0.4_Ti_0.2_O_2_-950 and Na_0.8_Ni_0.4_Mn_0.4_Ti_0.2_O_2_-850 materials are promising cathodes for SIBs with a long operation life and fast-charge performance, respectively.

### 3.3. Structural Evolution

**Figure 5:**
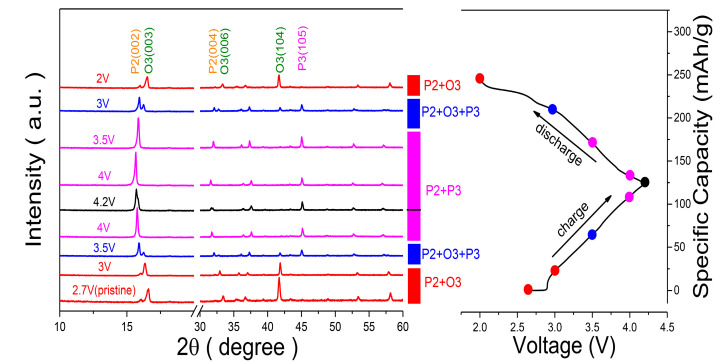
Ex-situ XRD patterns of 8442-950 electrode charged and discharged at different voltages.

To understand the relationship between structure evolution and the superior cycle stability of the P2/O3 Na_0.8_Ni_0.4_Mn_0.4_Ti_0.2_O_2_-950 electrode, ex-situ XRD measurements were performed in the first charge/discharge process. As demonstrated in Figure 5, when charging starts, the (003) diffraction peaks of O3 phases shift gradually to a lower 2θ angle, while the (104) peaks shift to a higher 2θ angle, suggesting the expansion of the interlayer distance and the shrinkage of the inter-planner distance, respectively. With further charging, new (003) peaks belonging to P3 phases emerged at a lower angle, and the peak’s intensity increased gradually, which demonstrates a two-phase reaction from O3 to P3 structure. Then, the (003) peaks of the P3 phase shift continuously to a lower angle until the charging ends, indicating a solid-solution reaction. Upon discharge, the P3 phase transforms back to the O3 phase with an opposite phase evolution, indicating the phase transition processes are reversible. Due to the low proportion of the P2 phase in the 8442-950 electrode and the overlap of (002) peaks (P2 phase) and (003) peaks (P3 phase), it is hard to assess accurately the complete structural evolution of the P2 phase and O3 phase. Nonetheless, when charging to 4.2 V, the position of the (004) peak belonging to the P2 phase shifts towards to lower angle compared to that of the initial state. Then it shifted back to the initial position at the end of the discharge. In addition, the high-voltage plateau is absent in charge-discharge curves, indicating the phase transition of P2-O2/OP4/Z is suppressed or delayed. Therefore, we speculate the P2 phase shows a solid-solution reaction upon Na^+^ (de)intercalation. These ex-situ XRD results indicate that the 8442-950 cathode shows a reversible phase transition of P2/O3-P2/P3, which facilitates excellent cycling and rate performance.

## 4. Conclusions

In summary, we have investigated the effect of Ti substitution and synthesis temperature on the phase ratio of P2/O3 and the Na storage performance of Na_0.8_Ni_0.4_Mn_0.6_O_2_. We found that Ti substitution and high synthesis temperature enable the formation of the O3-type structure and suppress the appearance of the P2-type structure, providing a feasible strategy to tune the proportion of P2/O3 phases and improve the electrochemical properties. The as-prepared O3-rich Na_0.8_Ni_0.4_Mn_0.4_Ti_0.2_O_2_-950 exhibits a specific capacity of 120 mAh/g and excellent long-cycle stability with a capacity retention of 84% (3 C, 700 cycles). The biphasic Na_0.8_Ni_0.4_Mn_0.4_Ti_0.2_O_2_-850 with elevated P2 characteristics shows a similar capacity and improved rate performance (65% capacity retention at the 5 C rate). Ex-situ XRD experiments indicate the Na_0.8_Ni_0.4_Mn_0.4_Ti_0.2_O_2_-950 cathode shows a reversible phase transition of P2/O3-P2/P3, which contributes to the excellent cycling performance. The present study provides rational guidance for the design of P2/O3 composite layered transition oxide cathodes with expected performance.

## Data Availability

The data presented in this study are available upon request from the corresponding author.

## Supplementary Materials

The following supporting information can be downloaded at: https://www.mdpi.com/article/10.3390/nano13081349/s1, Figure S1: XRD patterns and refinement results of (a) Na_0.8_Ni_0.4_Mn_0.6_O_2_-950, (b) Na_0.8_Ni_0.4_Mn_0.4_Ti_0.2_O_2_-950, (d) Na_0.8_Ni_0.4_Mn_0.2_Ti_0.4_O_2_-950, and (e) Na_0.8_Ni_0.4_Ti_0.6_O_2_-950. (c) and (f) the evolution of cell parameters *a*, *c*, and unit cell volume *V* of O3 and P2 structures with the increase in Ti-substitution.; Figure S2: XRD patterns and refinement results of (a) Na_0.8_Ni_0.4_Mn_0.4_Ti_0.2_O_2_-850, (b) Na_0.8_Ni_0.4_Mn_0.4_Ti_0.2_O_2_-900, (d) Na_0.8_Ni_0.4_Mn_0.4_Ti_0.2_O_2_-950, and (e) Na_0.8_Ni_0.4_Mn_0.4_Ti_0.2_O_2_-1000. (c) and (f) the evolution of cell parameters *a*, *c*, and unit cell volume *V* of O3 and P2 structures with the increase in synthesis temperature; Figure S3: The cycling performance of (a) 8442-850, (b) 8442-900, (c) 8442-950, (d) 8442-1000 electrode, respectively; Table S1: Structural parameters, atomic coordinates and occupancies of the Na_0.8_Ni_0.4_Mn_0.6_O_2_-950 obtained by refining XRD pattern; Table S2: Structural parameters, atomic coordinates and occupancies of the Na_0.8_Ni_0.4_Mn_0.4_Ti_0.2_O_2_-950 obtained by refining XRD pattern; Table S3: Structural parameters, atomic coordinates and occupancies of the Na_0.8_Ni_0.4_Mn_0.2_Ti_0.4_O_2_-950 obtained by refining XRD pattern; Table S4: Structural parameters, atomic coordinates and occupancies of the Na_0.8_Ni_0.4_Ti_0.6_O_2_-950 obtained by refining XRD pattern; Table S5: The designed chemical compositions of Na_0.8_Ni_0.4_Mn_0.6-x_Ti_x_O_2_-950 and calculated results obtained by refining XRD patterns; Table S6: Structural parameters and atomic coordinates and occupancies of the Na_0.8_Ni_0.4_Mn_0.4_Ti_0.2_O_2_-850 obtained by refining XRD pattern; Table S7: Structural parameters and atomic coordinates and occupancies of the Na_0.8_Ni_0.4_Mn_0.4_Ti_0.2_O_2_-900 obtained by refining XRD pattern; Table S8: Structural parameters and atomic coordinates and occupancies of the Na_0.8_Ni_0.4_Mn_0.4_Ti_0.2_O_2_-1000 obtained by refining XRD pattern; Table S9: The designed chemical compositions of Na_0.8_Ni_0.4_Mn_0.6-x_Ti_x_O_2_-950 and calculated results obtained by refining XRD patterns; Table S10: Stoichiometry of Na_0.8_Ni_0.4_Mn_0.4_Ti_0.2_O_2_-T (T = 850, 900, 950, 1000 °C, denoted as 8442-T) samples determined by ICP-AES; Table S11: The electrochemical performance comparison of P2/O3 biphasic layered oxide cathodes. References [34,37,39,40,41,42,43,44,45] are cited in Supplementary Materials.
