# Supplementary material for: Manipulating of P2/O3 Composite Sodium Layered Oxide Cathode through Ti Substitution and Synthesis Temperature
Source: Nanomaterials (Basel). 2023 Apr 12;13(8):1349. doi: 10.3390/nano13081349 (PMC10143052; doi:10.3390/nano13081349)
Supplement: Supplementary file 1 [file nanomaterials-13-01349-s001.zip › nanomaterials-2263941-supplementary.pdf]

*Supplementary Materials*

# Manipulating of P2/O3 Composite Sodium Layered Oxide Cathode through Ti Substitution and Synthesis Temperature

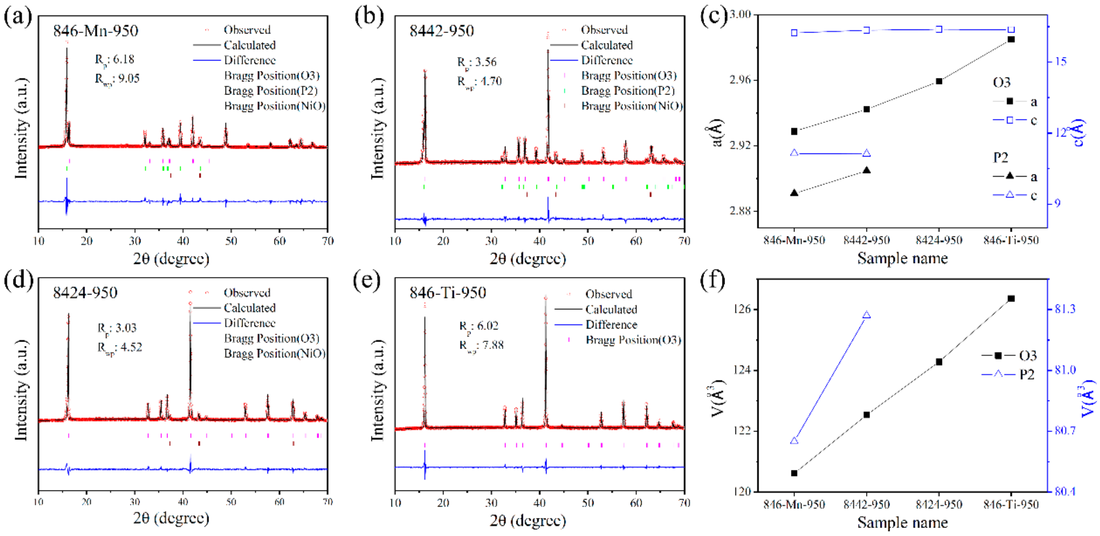


**Figure S1**. XRD patterns and refinement results of (a) $Na_{0.8}Ni_{0.4}Mn_{0.6}O_2$-950, (b) $Na_{0.8}Ni_{0.4}Mn_{0.4}Ti_{0.2}O_2$-950, (d) $Na_{0.8}Ni_{0.4}Mn_{0.2}Ti_{0.4}O_2$-950, and (e) $Na_{0.8}Ni_{0.4}Ti_{0.6}O_2$-950. (c) and (f) the evolution of cell parameters *a*, *c*, and unit cell volume *V* of O3 and P2 structures with the increase of Ti-substitution.

**Table S1**. Structural parameters, atomic coordinates and occupancies of the $Na_{0.8}Ni_{0.4}Mn_{0.6}O_2$-950 obtained by refining XRD pattern (*R*-factors: *R*p=6.18%, *R*wp=9.05%)

| Phase content | Atom | Site | *x* | *y* | *z* | g | Lattice parameters |
|---|---|---|---|---|---|---|---|
| O3 (28.5 wt%) | Na | *3b* | 0 | 0 | 0.5 | 0.860(9) | Space group: R-3m *a*=2.9260(7)Å *c*=16.2274(7) Å |
| | Ni/Mn | *3a* | 0 | 0 | 0 | 0.4/0.6 | |
| | O | *6c* | 0 | 0 | 0.2665(12) | 1 | |
| P2 (67.8 wt%) | Na1 | *2b* | 0 | 0 | 0.25 | 0.374(10) | Space group: $P6_3/mmc$ *a*=2.8896(6)Å *c*=11.1361(2)Å |
| | Na2 | *2d* | 0.6667 | 0.3333 | 0.25 | 0.358(11) | |
| | Ni/Mn | *2a* | 0 | 0 | 0 | 0.4/0.6 | |
| | O | *4f* | 0.6667 | 0.3333 | 0.0816(8) | 1 | |

**Table S2**. Structural parameters, atomic coordinates and occupancies of the $Na_{0.8}Ni_{0.4}Mn_{0.4}Ti_{0.2}O_2$-950 obtained by refining XRD pattern (*R*-factors: *R*p=3.56%, *R*wp=4.70%)

| Phase content | Atom | Site | *x* | *y* | *z* | g | Lattice parameters |
|---|---|---|---|---|---|---|---|
| O3 (74.9 wt%) | Na | *3b* | 0 | 0 | 0.5 | 0.809(18) | Space group: R-3m *a*=2.9417(3)Å *c*=16.3478(3)Å |
| | Ni/Mn/Ti | *3a* | 0 | 0 | 0 | 0.4/0.4/0.2 | |
| | O | *6c* | 0 | 0 | 0.2629(3) | 1 | |
| P2 (20.8 wt%) | Na1 | *2b* | 0 | 0 | 0.25 | 0.242(13) | Space group: $P6_3/mmc$ *a*= 2.9050(1)Å *c*=11.1151(5)Å |
| | Na2 | *2d* | 0.6667 | 0.3333 | 0.25 | 0.439(16) | |
| | Ni/Mn/Ti | *2a* | 0 | 0 | 0 | 0.4/0.4/0.2 | |
| | O | *4f* | 0.6667 | 0.3333 | 0.0913(11) | 1 | |

**Table S3**. Structural parameters, atomic coordinates and occupancies of the $Na_{0.8}Ni_{0.4}Mn_{0.2}Ti_{0.4}O_2$-950 obtained by refining XRD pattern

| O3 phase | | | | | |
|---|---|---|---|---|---|
| Atom | Site | *x* | *y* | *z* | g |
| Na | *3b* | 0 | 0 | 0.5 | 0.773(18) |
| Ni/Mn/Ti | *3a* | 0 | 0 | 0 | 0.4/0.2/0.4 |
| O | *6c* | 0 | 0 | 0.2666(3) | 1 |

Space group: R-3m, *a*= 2.9594(3)Å, *c*= 16.3852(3) Å, phase fraction 96.8 wt%
*R*-factors: *R*p=3.03%, *R*wp=4.52%

**Table S4**. Structural parameters, atomic coordinates and occupancies of the $Na_{0.8}Ni_{0.4}Ti_{0.6}O_2$-950 obtained by refining XRD pattern

| O3 phase | | | | | |
|---|---|---|---|---|---|
| Atom | Site | *x* | *y* | *z* | g |
| Na | *3b* | 0 | 0 | 0.5 | 0.809(15) |
| Ni/Ti | *3a* | 0 | 0 | 0 | 0.4/0.6 |
| O | *6c* | 0 | 0 | 0.2679(3) | 1 |

Space group: R-3m, *a*= 2.9849(19) Å, *c*= 16.3748(2) Å, phase fraction 100 wt%
*R*-factors: *R*p=6.02%, *R*wp=7.88%

**Table S5**. The designed chemical compositions of $Na_{0.8}Ni_{0.4}Mn_{0.6-x}Ti_xO_2$-950 and calculated results obtained by refining XRD patterns

| **Sample name** | **designed composition** | **Temperature (℃)** | **Phase** | **Weight Frac. (%)** |
|---|---|---|---|---|
| 846-Mn-950 | $Na_{0.8}Ni_{0.4}Mn_{0.6}O_2$ | 950 | O3-$Na_{0.86}Ni_{0.4}Mn_{0.6}O_2$ | 28.5 |
| | | | P2-$Na_{0.73}Ni_{0.4}Mn_{0.6}O_2$ | 67.8 |
| | | | NiO | 3.7 |
| 8442-950 | $Na_{0.8}Ni_{0.4}Mn_{0.4}Ti_{0.2}O_2$ | 950 | O3-$Na_{0.81}Ni_{0.4}Mn_{0.4}Ti_{0.2}O_2$ | 74.9 |
| | | | P2-$Na_{0.68}Ni_{0.4}Mn_{0.4}Ti_{0.2}O_2$ | 20.8 |
| | | | NiO | 4.3 |
| 8424-950 | $Na_{0.8}Ni_{0.4}Mn_{0.2}Ti_{0.4}O_2$ | 950 | O3-$Na_{0.77}Ni_{0.4}Mn_{0.2}Ti_{0.4}O_2$ | 96.8 |
| | | | NiO | 3.2 |
| 846-Ti-950 | $Na_{0.8}Ni_{0.4}Ti_{0.6}O_2$ | 950 | $Na_{0.81}Ni_{0.4}Ti_{0.6}O_2$ | 100 |

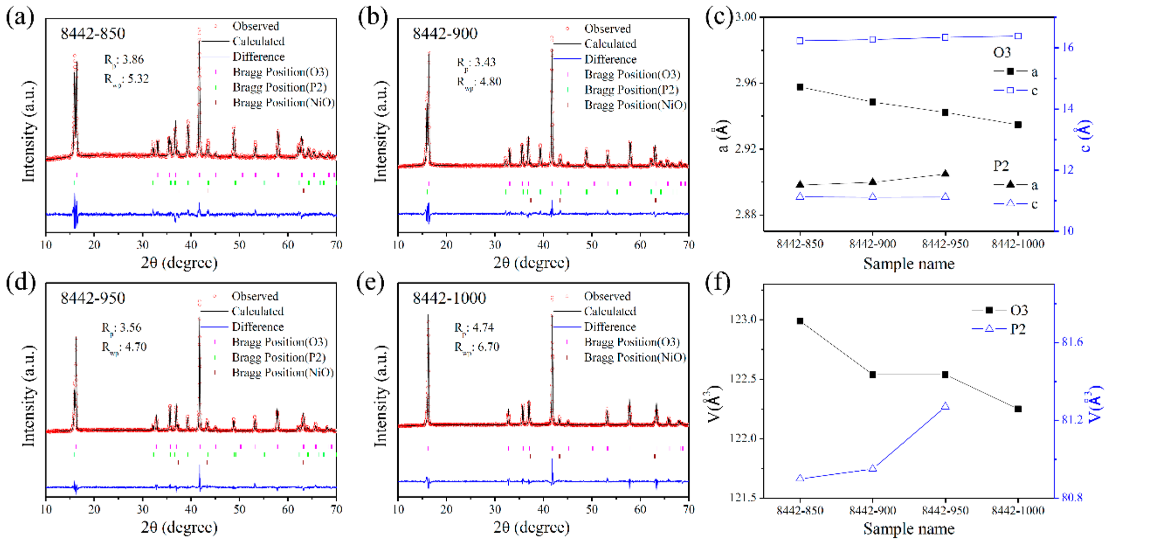


**Figure S2**. XRD patterns and refinement results of (a) $Na_{0.8}Ni_{0.4}Mn_{0.4}Ti_{0.2}O_2$-850, (b) $Na_{0.8}Ni_{0.4}Mn_{0.4}Ti_{0.2}O_2$-900, (d) $Na_{0.8}Ni_{0.4}Mn_{0.4}Ti_{0.2}O_2$-950, and (e) $Na_{0.8}Ni_{0.4}Mn_{0.4}Ti_{0.2}O_2$-1000. (c) and (f) the evolution of cell parameters *a*, *c*, and unit cell volume *V* of O3 and P2 structures with the increase of synthesis temperature.

**Table S6**. Structural parameters and atomic coordinates and occupancies of the $Na_{0.8}Ni_{0.4}Mn_{0.4}Ti_{0.2}O_2$-850 obtained by refining XRD pattern (*R*-factors: *R*p=3.86%, *R*wp=5.32%)

| Phase content | Atom | Site | *x* | *y* | *z* | g | Lattice parameters |
|---|---|---|---|---|---|---|---|
| O3 (59.0 wt%) | Na | *3b* | 0 | 0 | 0.5 | 0.858(10) | Space group: R-3m *a*=2.9579(5)Å, *c*=16.2345(5)Å |
| | Ni/Mn/Ti | *3a* | 0 | 0 | 0 | 0.4/0.4/0.2 | |
| | O | *6c* | 0 | 0 | 0.2608(4) | 1 | |
| P2 (37.1 wt%) | Na1 | *2b* | 0 | 0 | 0.25 | 0.274(12) | Space group: $P6_3/mmc$, *a*=2.8980(9)Å *c*=11.1272(4)Å |
| | Na2 | *2d* | 0.6667 | 0.3333 | 0.25 | 0.389(17) | |
| | Ni/Mn/Ti | *2a* | 0 | 0 | 0 | 0.4/0.4/0.2 | |
| | *4f* | 0.6667 | 0.3333 | 0.0983(9) | 1 | *4f* | |

**Table S7**. Structural parameters and atomic coordinates and occupancies of the $Na_{0.8}Ni_{0.4}Mn_{0.4}Ti_{0.2}O_2$-900 obtained by refining XRD pattern (*R*-factors: *R*p=3.43%, *R*wp=4.80%)

| Phase content | Atom | Site | *x* | *y* | *z* | g | Lattice parameters |
|---|---|---|---|---|---|---|---|
| O3 (67.8 wt%) | Na | *3b* | 0 | 0 | 0.5 | 0.827(18) | Space group: R-3m *a*=2.9489(3)Å *c*=16.2762(3)Å |
| | Ni/Mn/Ti | *3a* | 0 | 0 | 0 | 0.4/0.4/0.2 | |
| | O | *6c* | 0 | 0 | 0.2646(3) | 1 | |
| P2 (28.8 wt%) | Na1 | *2b* | 0 | 0 | 0.25 | 0.289(11) | Space group: $P6_3/mmc$, *a*= 2.9000(1)Å *c*=11.1191(4)Å |
| | Na2 | *2d* | 0.6667 | 0.3333 | 0.25 | 0.358(14) | |
| | Ni/Mn/Ti | *2a* | 0 | 0 | 0 | 0.4/0.4/0.2 | |
| | O | *4f* | 0.6667 | 0.3333 | 0.0935(10) | 1 | |

**Table S8**. Structural parameters and atomic coordinates and occupancies of the $Na_{0.8}Ni_{0.4}Mn_{0.4}Ti_{0.2}O_2$-1000 obtained by refining XRD pattern

O3 phase

| Atom | Site | *x* | *y* | *z* | g |
|---|---|---|---|---|---|
| Na | *3b* | 0 | 0 | 0.5 | 0.778(21) |
| Ni/Mn/Ti | *3a* | 0 | 0 | 0 | 0.4/0.4/0.2 |
| O | *6c* | 0 | 0 | 0.2653(3) | 1 |

Space group: R-3m, *a*= 2.93434(3) Å, *c*= 16.3860(3) Å, phase fraction 96.0 wt% *R*-factors: *R*p=4.74%, *R*wp=6.70%

**Table S9**. The designed chemical compositions of $Na_{0.8}Ni_{0.4}Mn_{0.6-x}Ti_xO_2$-950 and calculated results obtained by refining XRD patterns

| **Sample name** | **Designed composition** | **Temperature (°C)** | **Phase Weight Frac. (%)** | |
|---|---|---|---|---|
| 8442-850 | $Na_{0.8}Ni_{0.4}Mn_{0.4}Ti_{0.2}O_2$ | 850 | O3-$Na_{0.86}Ni_{0.4}Mn_{0.4}Ti_{0.2}O_2$ | 59.0 |
| | | | P2-$Na_{0.66}Ni_{0.4}Mn_{0.4}Ti_{0.2}O_2$ | 37.1 |
| | | | NiO | 3.9 |
| 8442-900 | $Na_{0.8}Ni_{0.4}Mn_{0.4}Ti_{0.2}O_2$ | 900 | O3-$Na_{0.83}Ni_{0.4}Mn_{0.4}Ti_{0.2}O_2$ | 67.8 |
| | | | P2-$Na_{0.65}Ni_{0.4}Mn_{0.4}Ti_{0.2}O_2$ | 28.8 |
| | | | NiO | 3.4 |
| 8442-950 | $Na_{0.8}Ni_{0.4}Mn_{0.4}Ti_{0.2}O_2$ | 950 | O3-$Na_{0.81}Ni_{0.4}Mn_{0.4}Ti_{0.2}O_2$ | 74.9 |
| | | | P2-$Na_{0.68}Ni_{0.4}Mn_{0.4}Ti_{0.2}O_2$ | 20.8 |
| | | | NiO | 4.3 |
| 8442-1000 | $Na_{0.8}Ni_{0.4}Mn_{0.4}Ti_{0.2}O_2$ | 1000 | O3-$Na_{0.78}Ni_{0.4}Mn_{0.4}Ti_{0.2}O_2$ | 96.0 |
| | | | NiO | 4 |

**Table S10**. Stoichiometry of $Na_{0.8}Ni_{0.4}Mn_{0.4}Ti_{0.2}O_2$-T (T=850, 900, 950, 1000 °C, denoted as 8442-T) samples determined by ICP-AES.

| Sample name | Na | Ni | Mn | Ti |
|---|---|---|---|---|
| 8442-850 | 0.794 | 0.404 | 0.410 | 0.186 |
| 8442-900 | 0.782 | 0.403 | 0.406 | 0.191 |
| 8442-950 | 0.773 | 0.406 | 0.406 | 0.188 |
| 8442-1000 | 0.768 | 0.409 | 0.404 | 0.187 |

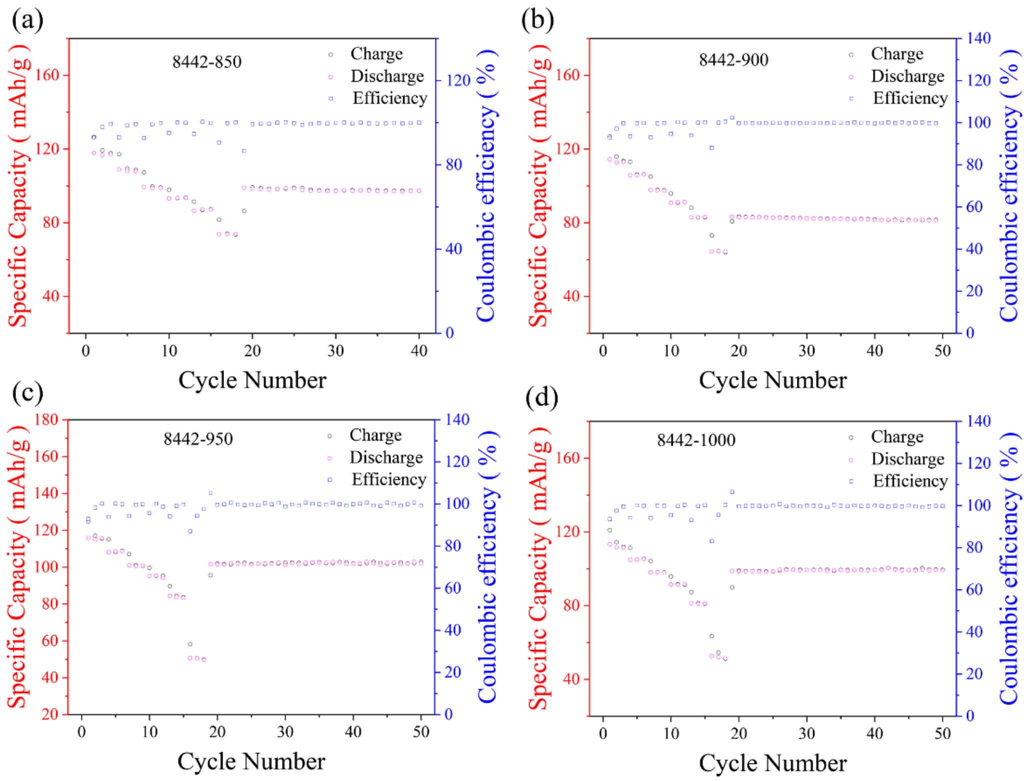


**Figure S3**. The cycling performance of (a) 8442-850, (b) 8442-900, (c) 8442-950, (d) 8442-1000 electrode, respectively.

Table S11. The electrochemical performance comparison of P2/O3 biphasic layered oxide cathodes.

| Materials | Voltage range (V) | Capacity (0.1C, mAh/g) | Rate | Cycling |
|---|---|---|---|---|
| $Na_{0.67}Ni_{0.33}Mn_{0.57}Sn_{0.1}O_2$ [1] | 2-4.3 | 155.2 | 66%, 150mA/g | 64%, 30cycle, 0.1C |
| $Na_{0.67}Fe_{0.425}Mn_{0.425}Mg_{0.15}O_2$ [2] | 1.5-4.2 | 146.1 | 40%, 2C | 95.7%, 50cycle, 1C |
| $Na_{0.67}Fe_{0.5}Mn_{0.46}Mg_{0.04}O_2$ [3] | 1.5-4.2 | 182(0.2C) | 61%, 1C | 63.9%, 70cycle,1C |
| $Li_{0.2}Na_{0.8}Ni_{0.33}Mn_{0.67}O_2$ [4] | 2-4.3 | 127.8 | 53%, 20C | 61.5%, 200cycle, 0.1C |
| P2-$Na_{2/3}MnO_2$-coated O3-$NaNi_{0.5}Mn_{0.5}O_2$ [5] | 2-4 | 141 | 73%, 15C | 85.3%, 150cycle, 1C |
| $Na_{0.85}Ni_{0.34}Mn_{0.33}Ti_{0.33}O_2$ [6] | 2.2-4.4 | 126.6 | 65%, 10C | 80.6, 200cycle, 1C |
| $Na_{0.7}Ni_{0.4}Mn_{0.4}Ti_{0.2}O_2$ [7] | 2-4.3 | 193 | 52%, 5C | 80.04%, 300cycle, 5C |
| $Na_{0.766}Ni_{0.33}Mn_{0.5}Fe_{0.1}Ti_{0.07}O_2$ [8] | 2.2-4.3 | 144(0.2C) | 64%, 10C | 75.4%, 500cycle, 5C |
| $Na_{7/9}Ni_{2/9}Mn_{4/9}Fe_{1/9}Mg_{1/9}Li_{1/9}O_2$ [9] | 2-4.4 | 170.5 | 62%, 10C | 71.8%, 500cycle, 5C |
| $Na_{0.8}Ni_{0.4}Mn_{0.4}Ti_{0.2}O_2$-950 | 2-4.2 | 120 | 54%, 5C | 84%, 700cycle, 3C |
| $Na_{0.8}Ni_{0.4}Mn_{0.4}Ti_{0.2}O_2$-850 (this work) | 2-4.2 | 120 | 65%, 5C | 74%, 700cycle, 3C |

**References**


1. Li, R.; Liu, Y.; Wang, Z.; Li, J. A P2/O3 biphasic cathode material with highly reversibility synthesized by Sn-substitution for Na-ion batteries. *Electrochim. Acta* **2019**, *318*, 14-22. https://doi.org/10.1016/j.electacta.2019.06.020.
2. Zhou, D.; Huang, W.; Lv, X.; Zhao, F. A novel P2/O3 biphase $Na_{0.67}Fe_{0.425}Mn_{0.425}Mg_{0.15}O_2$ as cathode for high-performance sodium-ion batteries. *J. Power Sources* **2019**, *421*, 147-155. https://doi.org/10.1016/j.jpowsour.2019.02.061.
3. Zhou, D.; Zeng, C.; Ling, D.; Wang, T.; Gao, Z.; Li, J.; Tian, L.; Wang, Y.; Huang, W. Sustainable alternative cathodes of sodium-ion batteries using hybrid P2/O3 phase $Na_{0.67}Fe_{0.5}Mn_{0.5-x}Mg_xO_2$. *J. Alloys Compd.* **2023**, *931*, 167567. https://doi.org/10.1016/j.jallcom.2022.167567.
4. Feng, J.; Luo, S.-h.; Cong, J.; Li, K.; Yan, S.; Wang, Q.; Zhang, Y.; Liu, X.; Lei, X.; Hou, P.-q. Synthesis and electrochemical properties of Co-free P2/O3 biphasic $Na_{1-x}Li_xNi_{0.33}Mn_{0.67}O_2$ cathode material for sodium-ion batteries. *J. Electroanal. Chem.* **2022**, *916*, 116378. https://doi.org/10.1016/j.jelechem.2022.116378.
5. Liang, X.; Yu, T.-Y.; Ryu, H.-H.; Sun, Y.-K. Hierarchical O3/P2 heterostructured cathode materials for advanced sodium-ion batteries. *Energy Storage Mate.* **2022**, *47*, 515-525. https://doi.org/10.1016/j.ensm.2022.02.043.
6. Yu, L.; Cheng, Z.; Xu, K.; Chang, Y.-X.; Feng, Y.-H.; Si, D.; Liu, M.; Wang, P.-F.; Xu, S. Interlocking biphasic chemistry for high-voltage P2/O3 sodium layered oxide cathode. *Energy Storage Mater.* **2022**, *50*, 730-739. https://doi.org/10.1016/j.ensm.2022.06.012.
7. Zhai, J.; Ji, H.; Ji, W.; Wang, R.; Huang, Z.; Yang, T.; Wang, C.; Zhang, T.; Chen, Z.; Zhao, W.; et al. Suppressing the irreversible phase transition from P2 to O2 in sodium-layered cathode via integrating P2- and O3-type structures. *Mater. Today Energy* **2022**, *29*, 101106. https://doi.org/10.1016/j.mtener.2022.101106.
8. Cheng, Z.; Fan, X.Y.; Yu, L.; Hua, W.; Guo, Y.J.; Feng, Y.H.; Ji, F.D.; Liu, M.; Yin, Y.X.; Han, X.; et al. A rational biphasic tailoring strategy enabling high-performance layered cathodes for sodium-ion batteries. *Angew. Chem.* **2022**, *134*, e202117728. https://doi.org/10.1002/ange.202117728.
9. Liang, X.; Sun, Y.K. A novel pentanary metal oxide cathode with P2/O3 biphasic structure for high-performance sodium-ion batteries. *Adv. Funct. Mater.* **2022**, *32*, 2206154. https://doi.org/10.1002/adfm.202206154.
